# Identification of the Bioavailable Peptidome of Chia Protein Hydrolysate and the In Silico Evaluation of Its Antioxidant and ACE Inhibitory Potential

**DOI:** 10.1021/acs.jafc.3c05331

**Published:** 2024-02-02

**Authors:** Alvaro Villanueva, Fernando Rivero-Pino, Maria E. Martin, Teresa Gonzalez-de la Rosa, Sergio Montserrat-de la Paz, Maria C. Millan-Linares

**Affiliations:** † Department of Food and Health, Instituto de la Grasa (IG-CSIC), Ctra. Utrera Km 1, 41013 Seville, Spain; ‡ Department of Medical Biochemistry, Molecular Biology, and Immunology, School of Medicine, University of Seville, Av. Sanchez Pizjuan s/n, 41009 Seville, Spain; § Instituto de Biomedicina de Sevilla, IBiS/Hospital Universitario Virgen del Rocio/CSIC/Universidad de Sevilla, Av. Manuel Siurot s/n, 41013 Seville, Spain; ∥ Department of Cell Biology, Faculty of Biology, University of Seville, Av. Reina Mercedes s/n, 41012 Seville, Spain

**Keywords:** ACE, bioactive peptides, DPPH, identification, protease, subtilisin

## Abstract

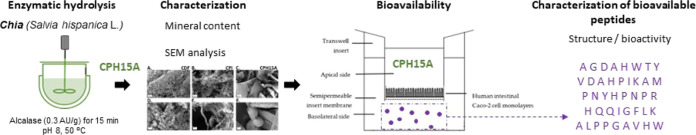

The incorporation of novel, functional, and sustainable foods in human diets is increasing because of their beneficial effects and environmental-friendly nature. Chia (*Salvia hispanica* L.) has proved to be a suitable source of bioactive peptides via enzymatic hydrolysis. These peptides could be responsible for modulating several physiological processes if able to reach the target organ. The bioavailable peptides contained in a hydrolysate obtained with Alcalase, as functional foods, were identified using a transwell system with Caco-2 cell culture as the absorption model. Furthermore, 20 unique peptides with a molecular weight lower than 1000 Da and the higher statistical significance of the peptide-precursor spectrum match (−10 log *P*) were assessed by in silico tools to suggest which peptides could be those exerting the demonstrated bioactivity. From the characterized peptides, considering the molecular features and the results obtained, the peptides AGDAHWTY, VDAHPIKAM, PNYHPNPR, and ALPPGAVHW are anticipated to be contributing to the antioxidant and/or ACE inhibitor activity of the chia protein hydrolysates.

## 1 Introduction

Seeds of chia (*Salvia hispanica* L.) are considered a nutritionally interesting food based on its content of fiber (ranging from 30 to 34%), oil (25–40%), and high-quality protein (18–24%). It was certified as a safe and novel food by the Food and Drug Administration (USA) in 2005, and in Europe, it was authorized to be marketed in 2009.^1,2^ Focusing on the protein fraction, the content is higher than in common food products like wheat, oats or rice, and comparable to other seeds used to produce protein isolates like hemp, peas, and chickpeas.^3^ The protein fraction, as an isolate, can be used as a source of peptides by enzymatic hydrolysis.

Research on bioactive peptides derived from chia demonstrate antibacterial,^4^ angiotensin converting enzyme (ACE) inhibitors,^5^ and antioxidant^6^ properties in vitro, among others. In addition, analysis employing cell culture^2^ or rats^7^ increase the evidence that these peptides can modulate the physiological status of human beings. At a nutritional level, protein cleavage into low molecular weight peptides improves their digestibility and can lead to loss of antigenicity, decreasing the immunoreactivity of the native protein.^8^ The enzymatic hydrolysis does not imply loss of nutritional value from the amino acids based on the mild reaction conditions.

The consumption of plant-based peptides, such as chia, is aligned with the Sustainable Development Goals (SDGs), such as “Good health and well-being”, “Responsible consumption and production”, and “Climate action”, based on the environmental-friendly character of plant crop production, and the health benefits associated with these products.^9^ However, there is little research concerning the bioavailability of peptides,^10^ and consequently, it is difficult to attribute actual health-promoting effects to these peptides. Their functionality is linked to their stability, which is dependent on the sequence. Their susceptibility to be degraded by gastrointestinal enzymes and their capacity or not to cross the intestinal barrier hinder their use as functional ingredient. In a mixture of peptides obtained by enzymatic hydrolysis, some of them are bioactive, and others are not. The comprehensive identification and characterization of the bioavailable peptides, that can be involved in controlling a disease, allows the producer to assert that the product might exert health-promoting properties. Protein hydrolysates are composed of a variety of molecules (amino acids, low molecular weight peptides or oligopeptides, proteins, as well as nonproteinaceous material) with very similar structures, impeding sometimes a full identification of the peptides and proteins mixture. To this regard, the development of bioinformatics tools, including in silico analysis can help to overcome this limitation, like Uniprot (KB) or Protein Data Bank for the acquisition of the amino acid sequences of proteins, and BIOPEP “Enzyme action” or ExPasy Peptide Cutter for in silico protein digestion. Currently, several studies are combining in vitro and in silico analyses to fully explore the potential of bioactive peptides as functional ingredients.^11,12^

Based on literature published, we hypothesized that peptides below <1000 Da would be bioavailable and bioactive, depending on their sequences. Many reports suggest that peptides obtained by Alcalase are bioavailable, but scarce information, to the author’s knowledge, has been published in relation to peptidome characterization subjected to cell culture-absorption conditions. Focusing on the antioxidant and ACE inhibitors character that peptides can have, especially from chia, there is still several gaps that requires research in the upcoming years.^13,14^ The aim of this study was to evaluate the bioavailable peptidome of a chia protein hydrolysate obtained with Alcalase. Peptides were identified from the original hydrolysate and from the fraction collected after subjecting the hydrolysate to Caco-2 cells in a transwell model. The peptides with a molecular weight <1000 Da with higher −10 log *P* value (corresponding to statistical significance of the peptide-precursor spectrum match) were subjected to several in silico analysis to characterize their bioactivity, as antioxidants and ACE inhibitors agents.

## 2 Materials and Methods

### 2.1 Chemicals and Samples

*S. hispanica* L. seeds were supplied by the Autonomous University of Nuevo Leon (Monterey, Mexico), from which the protein was isolated, as described previously^3^ The enzyme used was Alcalase 2.4 L, which was supplied by Novozymes (Novozymes, Bagsvaerd, Denmark). The rest of the chemicals (of analytical grade) were bought from Sigma Chemical Co. (St. Louis, MO, USA) and Merk (Merck, Darmstadt, Germany).

### 2.2 Hydrolysis of Chia Protein Concentrate

The chia protein concentrate (CPC), with a protein content of 82.85%, was dissolved at 7.5% w/v in distilled water to be hydrolyzed employing Alcalase as the catalyst. The enzyme-to-substrate ratio was 0.3 AU/g protein. One Anson activity unit (AU) is defined as the amount of enzyme that will release 1.0 μmol l-tyrosine from hemoglobin per min at 25 °C and pH 7.5. Temperature and pH were maintained at 50 and 8, respectively. The enzymatic reactions were carried out in a jacketed reactor, and after 15 min, the reaction mixture was heated at 85 °C for 15 min, to deactivate the protease. The product obtained was centrifuged at 9500*g* for 15 min, and the supernatant was collected. The chia protein hydrolysates (CPHs) obtained were designated as CPH15A.

### 2.3 Characterization of the Mineral Content by Inductively Coupled Plasma

A microwave-assisted digestion procedure was carried out to quantify the mineral content of the CPH15A. Reagents employed for digestion were HNO_3_ and H_2_O_2_, with a temperature of 200 °C. The analysis was done employing a plasma optical emission spectrometer [inductively coupled plasma (ICP), SpectroBlue].^15^

### 2.4 Molecular Weights by UHPLC

Molecular weights (MWs) were estimated by size exclusion chromatography (SEC) on an Acquity Arc equipped with a 2998PDA Detector, a Sample Manager FTN-R, and a Quaternary Solvent Manager-R (Acquity Arc, Waters Corporation, Milford, MA, USA) with a XBridge TM Protein BEH SEC 200 Å 2.5 μm 4.6 mm × 150 mm column. The standard proteins (Waters Corporation, Milford, MA, USA) were used to calibrate the column: uracil (0.112 kDa), ribonuclease A (13.7 kDa), albumin chicken egg white (44.2 kDa), and thyroglobulin bovine (669 kDa).^3^ A volume of 10 μL dissolved in 100 mM sodium phosphate buffer and 0.02% (w/v) sodium azide adjusted at pH 6.8 at a concentration of 1 mg/mL was injected. Protein elution was recorded by measuring its absorbance at 280 nm and analyzed with Empower 3 Personal GPC/SEC software (Waters Corporation, Milford, MA, USA).

### 2.5 Ultrastructural Characterization by Scanning Electron Microscopy Analysis

Ultrastructural characterization of the samples (defatted flour, concentrates, and CPH15A) was performed by using scanning electron microscopy (SEM). A description of the methodology can be found in Montserrat-de la Paz et al.,^16^

### 2.6 In Vitro Availability Using Caco-2 Cell Culture

Caco-2 cells were cultured in 12-well cell culture inserts in Dulbecco’s modified Eagle medium, supplemented with 10% heat-inactivated FBS and 1% penicillin/streptomycin. Cells were incubated at 37 °C under a modified atmosphere of 5% CO_2_ and given a fresh medium every 2–3 days. Cell monolayer integrity was monitored by transepithelial electrical resistance using a Millicell ERS-2 voltammeter (Millipore). Inserts were used for 2 weeks after seeding and had a resistance of at least 500 W/cm^2^. Inserts were transferred to 12-well plates and were initiated by replacing the medium with fresh medium containing CPH15A dissolved in distilled water at 1 mg/mL in the apical chamber. After 4 h, the content on the basolateral side was recovered in PBS.

### 2.7 Peptide Extraction, Purification, and Sequence Identification by LC-TIMS-MS/MS

Samples were acidified with 0.5% trifluoroacetic acid. The desalting and concentration step was performed with ZipTip C18 (Millipore), the digested samples were speed-vacuum-dried, and the total protein content was analyzed by a bicinchoninic acid assay. LC-TIMS-MS/MS was carried out using a nanoElute nanoflow ultrahigh-pressure LC system (Bruker Daltonics, Bremen, Germany) coupled to a timsTOF Pro 2 mass spectrometer equipped with a CaptiveSpray nanoelectrospray ion source (Bruker Daltonics). Details of the methodology can be found in Montserrat-de la Paz et al.,^16^ although in this case, the reference library is acquired from UniProt_proteome_Salvia-ssp_Feb22.

### 2.8 In Silico Analysis

The peptides chosen from the pool of peptides identified (criteria: from those with molecular weight <1000 Da); the first 20 with higher statistical significance of the peptide-precursor spectrum match (according to the −10 lg *P* value) from the bioavailable hydrolysate were subjected to in silico analyses: (a) ToxinPred software was employed to predict hydrophobicity, charge, isoelectric point, amphipathicity, steric hindrance, toxicity, and molecular weight of the peptides (https://webs.iiitd.edu.in/raghava/toxinpred/design.php);^17^ (b) PeptideRanker, which predicts probability to be bioactive (http://bioware.ucd.ie/~compass/biowareweb), giving an score from 0 to 1.0 at a threshold of 0.5;^18^ (c) AnOxPePred-1.0 was employed to predict the antioxidant (quantified by free radical scavenging and ion chelating scores) properties of peptides using convolutional neural network (https://services.healthtech.dtu.dk/service.php?AnOxPePred-1.0),^19^ (d) mAHTPred was used for predicting ACE inhibitors property of peptides using effective feature representation (http://thegleelab.org/mAHTPred/index.html). The assigned scores ranged from 0 to 1.0 at a threshold of 0.5;^20^ (e) Computational molecular stability: the web server PASTA 2.0 (http://protein.bio.unipd.it/pasta2/) was used to compute the tendency of peptide self-aggregation and the predicted amyloid-like structure (parallel/antiparallel aggregation) that were specific to the possible region at sequence, following the protocols from Li et al.,^21^ and Montserrat-de la Paz et al.,^16^

### 2.9 In Silico Digestion of Bioactive Peptides

The tool BIOPEP, which can be found at https://biochemia.uwm.edu.pl/biopep/rec_pro1.php?x=72&y=0, was employed to carry out an in silico gastrointestinal digestion of the peptides identified by entering the peptide sequences and predicting the potential sites cleaved by pepsin, chymotrypsin, and trypsin, aiming to predict the potential new species produced after digestive degradation. Then, these fragments were screened to see if they were ACE inhibitory or antioxidant sequences, according to the database.

### 2.10 Molecular Docking and Ligand-Interaction Visualization

Molecular docking was carried out to determine the binding affinity energy of the peptides with the ACE (Angiotensin Converting Enzyme) receptor. The X-ray crystal structure of the enzyme (PDB: 1O8A) was obtained from the RCSB PDB database (Protein Data Bank, http://www.rcsb.org/). Ligands and all the water molecules were removed from the receptor PDB file, while the polar hydrogen atoms were added, and the structure was minimized using UCSF Chimera software. The 3D structures of the two selected peptides were obtained with a USCF Chimera. Following that, the molecular structures of ACE and the peptides were converted to the PDBQT format with AutoDock Tools. The AGFR program was employed to determine the positions and sizes of the specific docking boxes for the selected sequences. Next, AutoDock Crank Pep was employed to perform docking analysis with the peptides. The potential best docking score determined was chosen and visualized via Biovia Discovery Studio Visualizer, as well as the two-dimensional (2D) and surface annotation of both ligand interactions with the protein.

### 2.11 Statistical Analysis

All values are presented as means ± standard deviations (SD). Data were evaluated using Graph Pad Prism version 9.1.2 (San Diego, CA, USA). The statistical significance of the antioxidant activity between the groups was assessed using a two-way analysis of variance (ANOVA), followed by Tukey’s multiple-comparison test. *P* values less than 0.05 were considered to be statistically significant.

## 3 Results and Discussion

### 3.1 Characterization

In previous reports, a characterization of the CPH15A was carried out, including its chemical composition, techno-functional properties (oil absorption capacity and capacity and stability of foaming and emulsifying), and cell-free bioactive properties (antioxidant and ACE inhibitors).^3^ The half maximal inhibitory concentration (IC_50_) value for inhibiting angiotensin converting enzyme (ACE) was 78.84 ± 1.21 μg/mL. The protein content of CPH15A was 75.03%, whereas the remaining fractions comprised fiber (11.2%), moisture (7.32%), and ash (6.45%). The amino acid profile of the hydrolysate was in line with the FAO/WHO/UNU nutritional recommendations for adults for essential amino acids. Among the nonessential ones, the majority are the negatively charged amino acids, glutamic acid and aspartic acid, in an amount of 178.3 and 84.5 mg/g protein, respectively, and the positively charged arginine (105.3 mg/g protein).^3^

CPH15A has been shown to be a promising ingredient to be employed to fortify foods, based on its improved properties. A more detailed characterization will provide a deeper insight into its nutritional properties and the molecules responsible for the bioactivity reported. In this work, the mineral content of CPH15A was evaluated. In Table 1, the results of the elemental analysis in mg/kg are reported. The element found in the highest amount was sodium, which is due to the alkali necessary to keep the pH constant during the hydrolysis process as well as the intrinsic content of chia. The content of sulfur mostly derives from the amount of cysteine and methionine quantified in the hydrolysate (40.32 mg/g protein).^2^ A balanced mineral content is important to treat the deficiency in the diet of these in the population, such as the potassium content, whereas it is also important to take into consideration the tolerable upper intake levels for essential minerals, not to lead to a nutritionally disadvantageous ingredient.

**Table 1:** Mineral Content of CPH15A by ICP^a^

element	CPH15A (mg/kg)
calcium	195.65 ± 0.02
cobalt	≤0.25
chromium	≤0.25
iron	46.94 ± 0.00
potassium	142.29 ± 0.02
magnesium	16.06 ± 0.00
manganese	0.25 ± 0.00
sodium	21195.65 ± 1.98
nickel	≤0.25
phosphorus	1711.96 ± 0.06
sulfur	12,500 ± 0.20
selenium	≤1.73
vanadium	≤0.25
zinc	3.71 ± 0.00

a Values expressed as mean content ± standard deviation (*n* = 3).

Kulczyński et al.^22^ reviewed the chemical composition and nutritional value of chia seeds, indicating phosphorus (860–919 mg/100 g), calcium (456–631 mg/100 g), potassium (407–726 mg/100 g), and magnesium (335–449 mg/100 g) as the most abundant ones. According to the bioprocess, the content of minerals is not comparable to the hydrolysate, as it is obtained from the protein isolate, and consequently, the amount of each fraction is not the same. However, this characterization demonstrates that CPH15A still supplies many minerals and is nutritionally interesting for human consumption.

**Figure 1:**
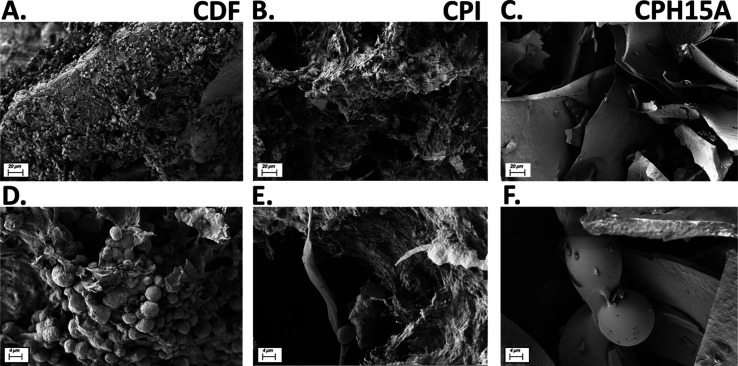
Surface characteristics of chia defatted flour (CDF), chia protein concentrate (CPC), and the CPC after 15 min of hydrolysis with Alcalase (CPH15A) by scanning electron microscopy (SEM) at two magnifications (Mag.). Upper SEM images (A–C) were taken at Mag = 345× and AV = 2.0 kV. Lower SEM images (D–F) were taken at Mag = 1.5k× and AV = 2.0 kV.

In addition, SEM analysis (Figure 1) showed how the surface morphology of the original chia sample changed after hydrolysis. It is observed that the protein presented in the chia defatted flour and CPI has been degraded into small fragments after the proteolytic action of Alcalase, cleaving several peptidic bonds and, as a consequence, leading to a reduction in particle size under the same SEM parameters (Mag = 345× and AV = 2.0 kV/Mag = 1.5k× and AV = 2.0 kV). These findings are similar to recently reported changes in the ultrastructural characterization of protein hydrolysates obtained from hemp or turtlegrass, which are additionally correlated with an increased solubility of the samples.^16,23^

### 3.2 Peptidome Profile

#### 3.2.1 Peptides Identified in CPH15A

**Figure 2:**
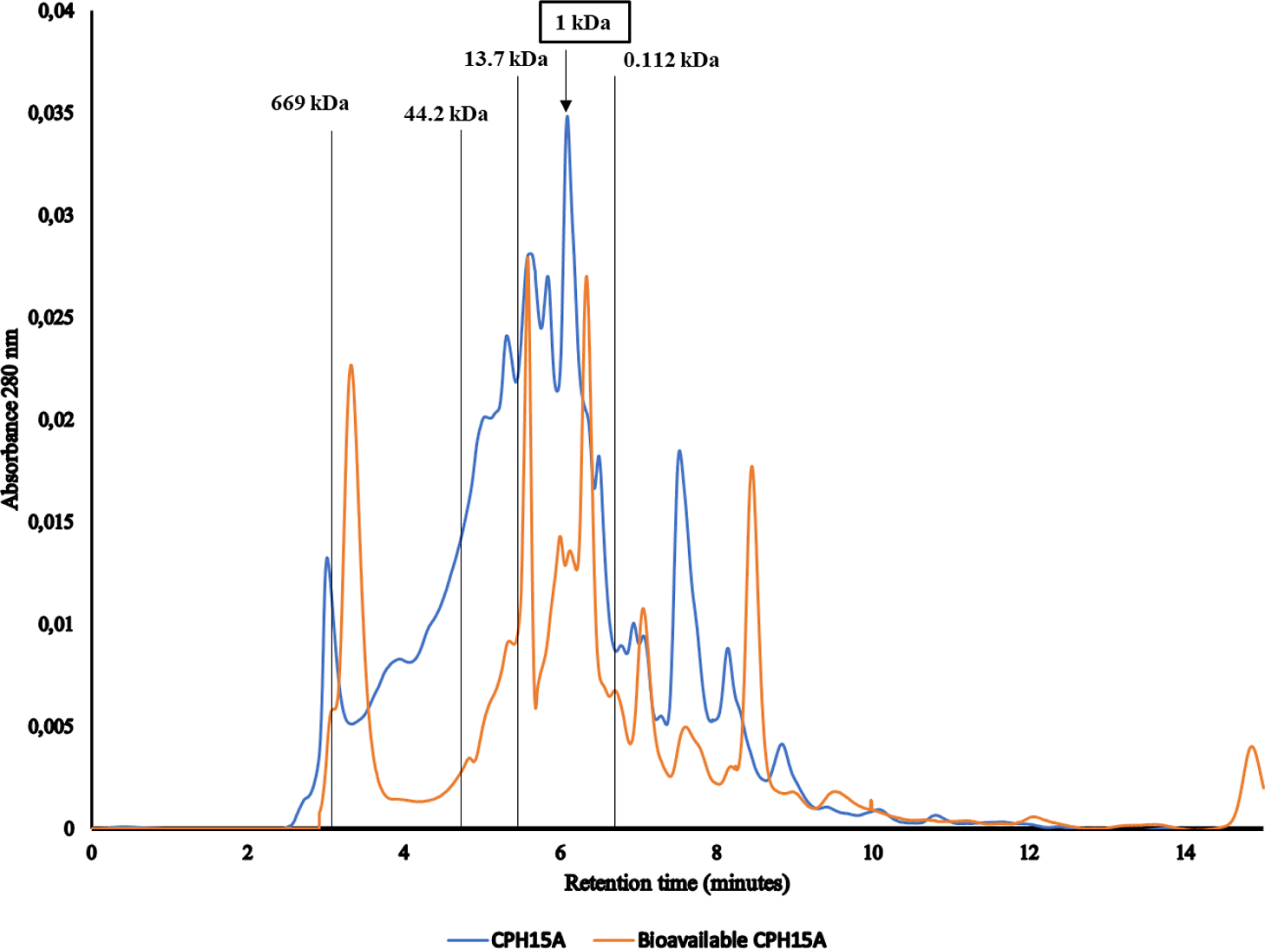
Molecular weight (MW) profiles by UHPLC chromatogram of CPH15A and bioavailable CPH15A.

The peptidome of CPH15A was fully characterized by LC-TIMS-MS/MS. In the original hydrolysate, a total of 1868 peptides were identified. The average length of all the peptides identified is 12 residues, but sequences up to 26 amino acids were identified. The average length of the 20 selected peptides was 15 residues. Only around 12% of the sample corresponded to <1000 Da peptides (based on the peptidome obtained, not from the SEC analysis). The degree of hydrolysis of the sample was determined to be 36.2%.^3^ The profile of peptides released after enzymatic hydrolysis depends mostly on the specificity of the protease and the duration of the reaction. As shown in Figure 2, the protein molecular profile by UHPLC of CPH15A and bioavailable CPH15A showed that the peptide size was both similar, around 3–0.2 kDa and 5.9–6.6 retention time, respectively. This result showed that enzymatic hydrolysis using Alcalase significantly produced bioavailable peptides. In this case, Alcalase shows subtilisin activity, which is an endopeptidase that is high-spectrum, nonspecific, and would cleave mainly hydrophobic amino acids.^24^

Due to the technique employed for the identification of peptides, the sequence of high-molecular-weight proteins such as globulins is not reported in the outcome of this analysis, but it encompasses only the peptide up to a certain amino acid length. In the scope of this manuscript, the focus is on peptides below 1000 Da, as they are expected to be bioactive^25,26^ because of their ability to enter cells. Chia is not a substrate as widely studied as other common protein sources, such as fish or milk.^27^ However, there is only recently published literature reporting the identification of peptides from chia. For instance, five sequence peptides (NNVFYPF, FNIVFPG, SRPWPIDY, QLQRWFR, and GSRFDWTR) were identified by Aguilar-Toalá et al.^5^ Similar to NNVFYPF, in the original hydrolysate, the peptides RNNVFYPFD, RNNVFYPFE, and RNNVFYPF were identified. Similarly, other peptides containing those identified by these authors were found in the hydrolysate under assessment and will be further discussed in the following sections. Further research is required to explore how different proteases would release a different profile of peptides from the same substrate, as this will determine their applicability as functional ingredient.

#### 3.2.2 Bioavailable Peptides Identified after the Cell Culture Assay

In the bioavailable hydrolysate, a total of 1182 peptides were identified, 63% of the original hydrolysate. The average length of all of the peptides identified is 10 residues. It must be taken into consideration two factors when comparing the hydrolysate and the bioavailable hydrolysate: (i) Caco-2 cells: these can differentiate into monolayers with a phenotype with many functions of the small intestinal villus epithelium. Many brush-border enzymes and transport proteins mediate the active transport or efflux of molecules in these cells;^28^ as a consequence, the peptides going through these cells would be likely subjected to some kind of modification, mainly cleavage. (ii) The limitation of the technique employed to identify the peptidome: the parameters would include the peptides identified only if found in a minimum amount. However, this limitation lacks relevance as, if not detected, their contribution to bioactivity is likely to be negligible.

Focusing on <1000 Da peptides, 81 sequences were identified both in the hydrolysate before and after being subjected to absorption simulation in the transwell system. These sequences are able to cross the intestinal barrier without suffering any kind of modification, as explained previously. For instance, of the three peptides similar to NNVFYPF,^5^ two (RNNVFYPFE and RNNVFYPF) were identified as bioavailable in our research, whereas RNNVFYPFD demonstrated that it did not cross the barrier or was not identified in a sufficient amount.

It has been previously described that the presence of the amino acid arginine at the terminal of the sequences might contribute to an increased resistance to gastrointestinal digestion since the digestive proteases are specific toward these amino acids.^29^ This statement is in line with the results obtained since 12 out of the 81 peptides (15% of the total) identified possess R in the N-terminal and 13 of them at the C-terminal (16%). Other bioactive peptides from plant sources, such as GPETAFLR, isolated from a protein hydrolysate from *Lupinus angustifolius* L., have arginine in their sequence, which may be one of the reasons for their anti-inflammatory potential.^30^ The peptide REGADFVR possesses two terminals with arginine. On top of that, several studies have reported the maintainance, loss, or gain of peptides after simulation of digestion using Alcalase, and this would depend on the sequences of the hydrolysate.^29,31^

Considering that the <1000 Da peptides identified in the bioavailable fraction are more likely to be bioactive, the 20 with higher statistical significance of the peptide-precursor spectrum match where subjected to in silico analyses to evaluate their bioactive potential. Out of these 20 peptides, 11 were also identified in the original hydrolysate, whereas the other 9 were not. The identification of peptides before and after being subjected to absorption-simulator models is relevant to evaluating if the peptides exerting bioactivity are maintained or modified, and it gives an answer to whether encapsulation might be needed to stabilize the peptides as nutraceuticals before being incorporated into a food matrix.^32^

### 3.3 In-Silico Analyses of Bioavailable Peptides

In Table 2, the physical-chemical properties (i.e., net charge, isoelectric point (pI), hydrophobicity, steric hindrance, and amphipathicity) and in Table 3 the likelihood to be bioactive and the outcome from bioactivity prediction tools (i.e., free radical scavenger, chelation score, and ACE inhibitor property) and the in silico digestion products of each peptide are shown. According to the outcome of the ToxinPred software analysis, none of the peptides are predicted to be toxic. Regarding the physic-chemical parameter, and as expected from an Alcalase-aided hydrolysis, the hydrophobicity shows in most of the peptides promising values and, as shown in the literature, can be positively correlated with bioactivity.

**Table 2:** Physical-Chemical Characterization of the Selected Peptide Sequences Identified in Bioavailable Chia Protein Hydrolysate (CPH15A) Based on in silico Analyses^a^

peptide	–10 lg *P*	molec. weight	res. length	hydrophobicity	steric hindrance	amphipathicity	charge	pI
**IVDHSGQTM**	55.43	986.45	9	–0.06	0.60	0.30	–0.50	5.09
**VVDHSGQTM**	55.21	972.43	9	–0.08	0.60	0.30	–0.50	5.09
**HGPIKLH**	52.44	800.47	7	–0.08	0.42	0.94	2.00	9.11
**AGDAHWTY**	51.82	919.38	8	–0.03	0.53	0.18	–0.50	5.09
**TNAPRLTF**	49.87	918.49	8	–0.18	0.58	0.31	1.00	10.11
**KNLDHPTSA**	48.76	981.49	9	–0.29	0.52	0.57	0.50	7.09
**VDAHPIKAM**	47.92	980.51	9	–0.03	0.56	0.57	0.50	7.09
**FSEDNVKVG**	47.77	993.48	9	–0.17	0.69	0.55	–1.00	4.38
**YTNAPRLT**	47.67	934.49	8	–0.25	0.58	0.31	1.00	9.10
**PNYHPNPR**	46.79	993.48	8	–0.45	0.50	0.49	1.50	9.10
**AEKGTLFPN**	45.33	975.50	9	–0.12	0.60	0.55	0.00	6.35
**SHKLPILN**	44.14	920.54	8	–0.09	0.51	0.64	1.50	9.11
**KQGDVIAIR**	43.37	998.59	9	–0.21	0.68	0.82	1.00	9.10
**HQQIGFLK**	42.94	969.54	8	–0.11	0.58	0.95	1.50	9.11
**VKEPVFSF**	42.61	951.51	8	0.03	0.63	0.62	0.00	6.35
**YTNAPRL**	41.75	833.44	7	–0.26	0.58	0.35	1.00	9.10
**ALPPGAVHW**	41.33	946.50	9	0.17	0.46	0.16	0.50	7.10
**NDGDAPLTY**	41.26	964.41	9	–0.15	0.62	0.00	–2.00	3.57
**HRQPQLN**	40.06	891.47	7	–0.53	0.53	0.91	1.50	10.11
**DAREPSYR**	39.62	992.47	8	–0.61	0.61	0.77	0.00	6.42

a Peptides were subjected to calculation via http://pepcalc.com/, where the net charge at neutral pH was calculated. Meanwhile, peptide solubility in pure water was estimated on this web server based on the combined results of the isoelectric point (pI), the number of charged residues, and the peptide length. Peptides were subjected to calculation via https://webs.iiitd.edu.in/raghava/toxinpred/design.php/, where the hydrophobicity, steric hindrance, and amphipathicity were calculated.

**Table 3:** Bioactivity Prediction (i.e., Free Radical Scavenger, Chelation Score, and ACE Inhibitors Property) and Digestion of the Selected Peptide Sequences Identified in Bioavailable Chia Protein Hydrolysate (CPH15A) Based on In Silico Analyses^a^

peptide	–10 lg *P*	likelihood of being bioactive	free radical scavenger score	chelation score	mAHTPred	BIOPEP SGID	active fragments
IVDHSGQTM	55.43	0.163	0.411	0.267	0.13	IVDH-SGQTM	no
VVDHSGQTM	55.21	0.133	0.442	0.257	0.10	VVDH-SGQTM	no
HGPIKLH	52.44	0.450	0.420	0.251	0.27	H-GPIK-L-H	no
AGDAHWTY	51.82	0.605	0.532	0.217	0.11	AGDAH-W-TY	TY (antioxidant)
TNAPRLTF	49.87	0.499	0.358	0.229	0.23	TN-APR-L-TF	TF (ACE inhibitor)
KNLDHPTSA	48.76	0.157	0.403	0.258	0.24	K-N-L-DH-PTSA	no
VDAHPIKAM	47.92	0.517	0.451	0.280	0.51	VDAH-PIK-AM	no
FSEDNVKVG	47.77	0.256	0.327	0.200	0.09	F-SEDN-VK-VG	VK, VG (ACE inhibitors)
YTNAPRLT	47.67	0.473	0.392	0.212	0.18	Y-TN-APR-L-T	no
PNYHPNPR	46.79	0.751	0.603	0.283	0.85	PN-Y-H-PN-PR	PR (ACE inhibitor)
AEKGTLFPN	45.33	0.392	0.377	0.211	0.15	AEK-GTL-F-PN	no
SHKLPILN	44.14	0.440	0.356	0.267	0.54	SH-K-L-PIL-N	no
KQGDVIAIR	43.37	0.238	0.271	0.180	0.11	K-QGDVIAIR	no
HQQIGFLK	42.94	0.547	0.386	0.234	0.10	H-QQIGF-L-K	no
VKEPVFSF	42.61	0.442	0.354	0.204	0.75	VK-EPVF-SF	VK, SF (ACE inhibitors)
YTNAPRL	41.75	0.297	0.421	0.223	0.76	Y-TN-APR-L	no
ALPPGAVHW	41.33	0.656	0.471	0.257	0.83	AL-PPGAVH-W	no
NDGDAPLTY	41.26	0.382	0.384	0.241	0.12		
HRQPQLN	40.06	0.301	0.406	0.266	0.36	N-DGDAPL-TY	TY (antioxidant)
DAREPSYR	39.62	0.310	0.410	0.249	0.17	DAR-EPSY-R	no

a The likelihood of the peptides being bioactive was evaluated by PeptideRanker (http://bioware.ucd.ie/~compass/biowareweb), a server to predict bioactive peptides based on a novel N to-1 neural network, by giving scores ranging from 0 to 1. A higher score indicated a greater likelihood of the peptide being bioactive. AnOxPePred tool (http://services.bioinformatics.dtu.dk/service.php?AnOxPePred-1.0) uses deep learning to predict the antioxidant properties (quantified by free radical scavenging and ion chelating scores) of peptides by giving scores ranging from 0 to 1. BIOPEP SGID refers to BIOPEP-simulated gastrointestinal digestion. Active fragments are those among the products that originated and are considered ACE inhibitory fragments, according to the information contained in the database.

Focusing on the value obtained from the antioxidant peptide predictor, the sequences PNYHPNPR, AGDAHWTY, ALPPGAVHW, and VDAHPIKAM have the highest free radical scavenger score, and in addition, PNYHPNPR and VDAHPIKAM are those with the highest chelation score. This result offers an idea of the peptides of CPH15A that could highly contribute to the antioxidant activity of the hydrolysate,^3^ as well as the immunomodulatory^2^ activity since the hydrolysate proved to reduce reactive oxygen species and nitrite output as well as proinflammatory cytokine secretion and enhance the expression and release of anti-inflammatory cytokines.

In addition, concerning the likelihood of being bioactive, according to Mooney et al.,^33^ a score >0.5 strongly suggests that the peptide is bioactive based on its molecular characteristics. It is interesting to see that the peptides that meet this characteristic are the same ones that reported high levels of antioxidant capacity according to the AnOxPred tool, in addition to the HQQIGFLK sequence, which, despite presenting a value higher than the established bioactivity threshold, does not report very high values of antioxidant activity in the tool used. This increases the evidence that these peptides are responsible for the bioactivity of the hydrolysate and could reach the target organs in vivo, as they have proved to be bioavailable in the cell model employed in this research.

Based on the physico-chemical characteristics of the individual peptides, it has been suggested that low steric hindrance values and high amphipathicity could contribute to increasing the bioactivity because they help stabilize the interaction of the peptide with the target compound.^34,35^ The peptides with the highest amphipathicity were HGPIKLH, HQQIGFLK, and HRQPQLN, which also showed adequate values in the other prediction tools employed and consequently could be also considered bioactive peptides from chia seeds.

León-Madrazo and Segura Campos^4^ carried out an in silico prediction of antimicrobial, antibiofilm, and antioxidant peptides from chia (*S. hispanica* L.) and indicated, in terms of antioxidant capacity, that the fragment LK is biologically relevant. This fragment was found in the peptide HQQIGFLK in the hydrolysate obtained, which was reported as bioactive according to the prediction tool aforementioned. In a similar way, Grancieri et al.,^36^ carried out molecular docking with many chia-derived peptides (e.g., TGPSPTAGP, PAPGGGTH, SPKDLALPPGALPPV, and HYGGPPGG) in order to elucidate their metal chelation or hydrogen/electron donor ability, as well as their potential interaction with inflammation and atherosclerosis markers. Although these peptides were not identified in our hydrolysate, some matching sequences were found, the most extensive being ALPPGA, which appears in SPKDLALPPGALPPV, from the peptides of Grancieri et al.^36^ and in ALPPGAVHW of bioavailable CPH15A, demonstrating that the peptides that make up chia proteins are a promising source of bioactive peptides. A peptide can exert antioxidant activity according to the residues in its sequence and how the functional groups of these amino acids can interact with the reactive oxygen species and counteract their effect. It has been proposed that the presence of valine (V) at the C-terminal or having tyrosine (Y) in any of the terminals^37^ is related to higher antioxidant properties. These features are reported in AGDAHWT**Y**, **Y**TNAPRL, **Y**TNAPRLT, and NDGDAPLT**Y**; indeed, the first one is proposed as highly antioxidant according to the tools, and the rest show an antioxidant score of around 0.4. In the same line, methionine (M) and histidine (H) are considered key contributors in the oxidative stress response because of their reactive oxygen species scavenger properties. These amino acids are also found in some of the peptides identified, including PNY**H**PNPR, ALPPGAV**H**W, and VDA**H**PIKA**M**, and some others are not predicted to be as highly antioxidant as these two, but that could be adding activity, even if to a lesser extent.

It is really interesting to see the correlation of these results with the outcomes from the prediction of ACE inhibitor peptides. A threshold of 0.5 is described by the authors of the prediction tool, and consequently, the following sequences could be considered ACE inhibitors: VDAHPIKAM, PNYHPNPR, SHKLPILN, VKEPVFSF, YTNAPRL, and ALPPGAVHW, 30% of the 20 selected peptides. The two reported as the most active ACE inhibitors would be PNYHPNPR and ALPPGAVHW, which were also reported as highly antioxidant. These two peptides were found in the hydrolysate before and after being subjected to the bioavailability assay, indicating their resistance to absorption conditions and suggesting their potential to achieve the bloodstream and exert their bioactivity in the target organs. The multifunctionality of peptides obtained from Alcalase has been already reported for chia^5^ as well as for other substrates such as insects,^38^ and it implies an increase of the value of the manufactured protein hydrolysates. In fact, it has been reported that ACE inhibitor peptides are usually related to the presence of certain amino acids, including proline (P), phenylalanine (F), tyrosine (Y), or tryptophan (W) at the C-terminal, and valine (V), leucine (L), or isoleucine (I) at the N-terminal.^39^ The bioactive potential of chia peptides, as described by Aguilar-Toalá et al.,^5^ revealed that the following sequences had the lowest energy score for ACE, suggesting their ACE inhibitory activity: NNVFYPF, FNIVFPG, SRPWPIDY, QLQRWFR, GSRFDWTR, DFKF, DLRF, FKAF, FRSF, and QFRF. Some similarities can be found among this pool of peptides and those identified in the hereby described hydrolysate. For instance, the SF terminal from the peptide FRSF is also found in the one described as highly antioxidant VKEPVF**SF**, as is the motif VF from FNI**VF**PG, increasing the evidence that this sequence is highly participating in the antioxidant activity described for the hydrolysate,^3^ or, in the same line, the motif PG can be found in the sequence ALP**PG**AVHW, with a value in the tool of 0.83.

Concerning the isoelectric point of peptides, it has been described that low values are associated with increased antioxidant activity, whereas no evident correlation was observed between this parameter and the ACE inhibitory properties of peptides.^40^ Among the four peptides predicted to be highly antioxidant, the sequence AGDAHWTY is reported to have the lowest isoelectric point, whereas ALPPGAVHW is the one with the lowest steric hindrance. Although verification of bioactivity should be carried out employing synthetic peptides, these analyses show the potential of in silico tools in the economic and fast characterization of bioactive peptides.^41^ The isoelectric point and amphiphacity of peptides are also important, for instance, when considering their antioxidant potential if used in an emulsion, as they can be at the interface between oil and water, stabilizing it.^42^

Taking into account all the parameters discussed, the peptides AGDAHWTY and VDAHPIKAM show promising results in terms of potential antioxidant activity. These peptides were not identified in the original hydrolysate though. This implies that these peptides are released from the hydrolysate due to the activity of the cells, and their concentration has highly increased so that it is quantifiable in the bioavailable fraction. On top of these two, PNYHPNPR and ALPPGAVHW are also proposed as peptides in CPH15A exerting high antioxidant and ACE inhibitor properties, and in this case, they are present in the original hydrolysate, as previously discussed.

On the other hand, the identified peptides were also in silico digested using the BIOPEP tool, as shown in the two last columns of Table 3. Only a few fragments theoretically released after the action of the proteases have been reported as antioxidant or ACE inhibitory sequences, considering the database employed, indicating that these would potentially exert activity after being orally ingested.

Beyond these factors regarding the primary structure of peptides, understanding the advanced conformation of peptides (i.e., secondary structure) also provides an insight into defining bioactive peptides and their role in antioxidant functionality.^21^ Herein, as computed by the PASTA 2.0 server (Table 4), 19 of 20 peptide sequences (95% in quantity) derived from CPH15A had the entire probability of rendering a random coil without any contribution to either the α-helix or the β-strand, and only 1 (KQGDVIAIR) of 20 sequences (5% in quantity) enabled the formation of the β-strand in simulation. Overall, through our computation, the amyloids and self-aggregation were not substantially favored by these peptide monomers of interest, regardless of their hydrophobicity.

**Table 4:** Secondary Structure Prediction of the Selected Peptide Sequences Identified in Bioavailable Chia Protein Hydrolysate (CPH15A) Based on In Silico Analyses^a^

peptide	–10 lg *P*	self-aggregation-prone region & amyloids	disorder probability (%)	probability in secondary structure (%)		
				α-helix	β-strand	coil
**IVDHSGQTM**	55.43	1–4 (NI)	100			100
**VVDHSGQTM**	55.21	1–4 (NI)	100			100
**HGPIKLH**	52.44	4–7 (NI)	100			100
**AGDAHWTY**	51.82	4–8 (NI)	100			100
**TNAPRLTF**	49.87	5–8 (NI)	100			100
**KNLDHPTSA**	48.76	2–5 (NI)	100			100
**VDAHPIKAM**	47.92	1–4 (NI)	100			100
**FSEDNVKVG**	47.77	5–8 (NI)	100			100
**YTNAPRLT**	47.67	1–4 (NI)	100			100
**PNYHPNPR**	46.79	2–5 (NI)	100			100
**AEKGTLFPN**	45.33	4–7 (NI)	100			100
**SHKLPILN**	44.14	4–7 (NI)	100			100
**KQGDVIAIR**	43.37	5–8 (PA)	100		44.44	55.56
**HQQIGFLK**	42.94	4–7 (NI)	100			100
**VKEPVFSF**	42.61	5–8 (NI)	100			100
**YTNAPRL**	41.75	1–4 (NI)	100			100
**ALPPGAVHW**	41.33	6–9 (NI)	100			100
**NDGDAPLTY**	41.26	6–9 (NI)	100			100
**HRQPQLN**	40.06	1–4 (NI)	100			100
**DAREPSYR**	39.62	4–8 (NI)	100			100

a The web server PASTA 2.0 (http://protein.bio.unipd.it/pasta2/) was implicated in computing the tendency of peptide self-aggregation specific to the possible region at sequence (with the recorded number starting from the N-terminus). For peptide discrimination, the optimal thresholds were switched to top = 1 and energy <−5 PEU (1 PEU (Pasta Energy Unit) = 1.192 kcal/mol). NI: no amyloid predicted; PA: parallel aggregation computed. The probability of intrinsic disorder and the portion of estimated secondary structure that complements the aggregation data were also reported.

**Figure 3:**
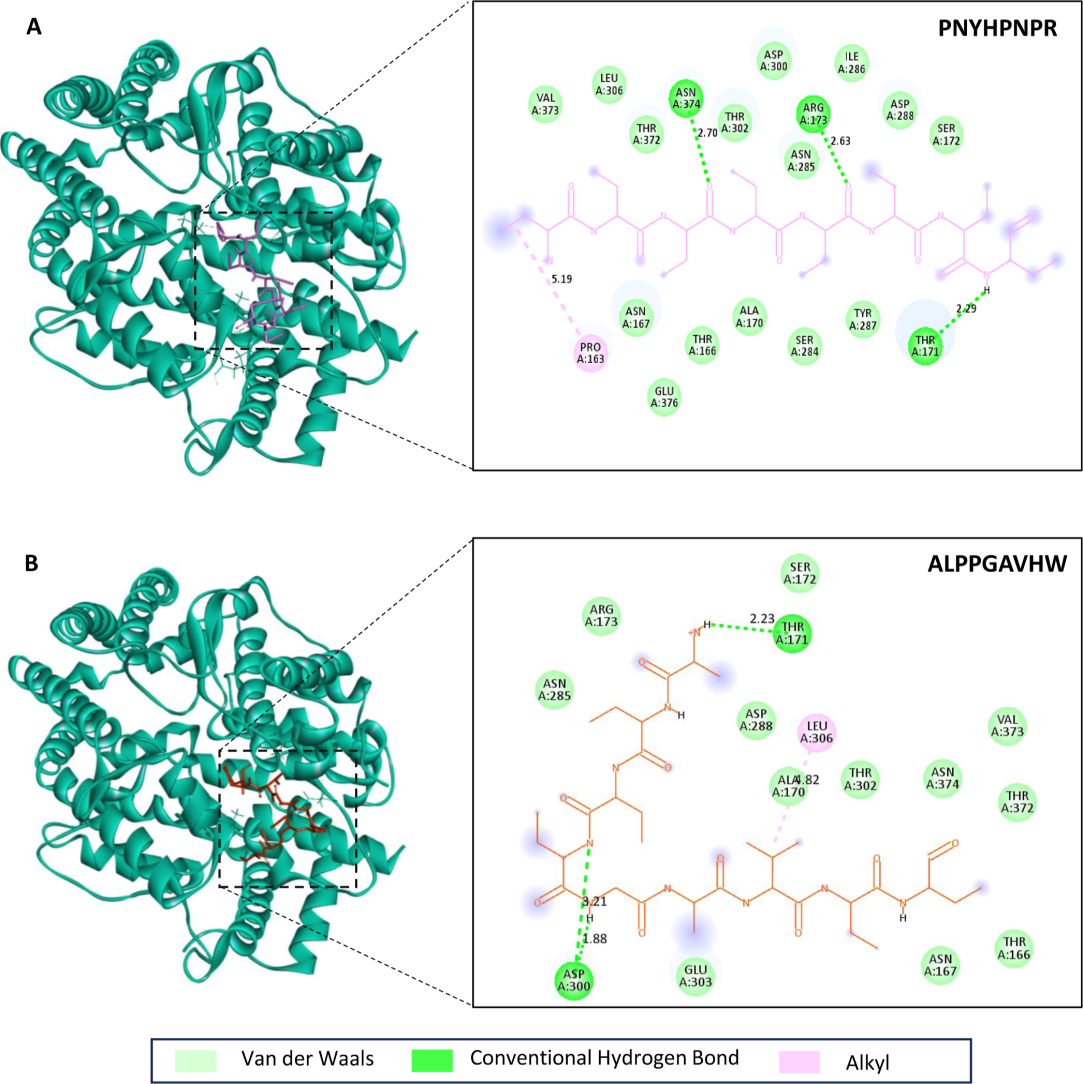
Visualization of the peptides-receptor binding (left) and 2D interaction diagram análisis (right) using Biovia Discovery Studio Visualizer. (A) ACE-PNYHPNPR binding site and their interactions with ACE and (B) ACE-ALPPGAHVW binding site and their interactions with ACE.

Lastly, the two peptides proposed as the most active were analyzed by molecular docking with the ACE enzyme. The affinity of the PNYHPNPR peptide with the ACE was −17.8 kcal/mol, whereas it was −19.5 kcal/mol for the sequence ALPPGAVHW, which is lower than values commonly found in recent literature for peptides interacting with this enzyme,^43,44^ indicating the likelihood of these peptides to interact with ACE and exert ACE inhibitory activity. In Figure 3, the 3D representation of the binding site and interactions of these two peptides with the ACE is shown, together with the graphical representation of the interaction occurring, where it can be observed that diverse types of interactions are occurring, leading to a stable complex, supporting the hypothesis that chia-derived peptides are responsible for the inhibition of ACE.

To the authors’ knowledge, the hereby identified peptides proposed as antioxidant and ACE inhibitor peptides stemming from a CPH obtained with Alcalase have not been identified previously. In the research field of bioactive peptides, there is still a long way to go. Several limitations (e.g., the in vivo effect of a protein hydrolysate accounts for the whole mixture of peptides and, consequently, the possible synergy effects among peptides) have to be addressed, and improvements in the techniques (peptidomics, in silico prediction tools, etc.) are needed in order to clearly characterize a protein hydrolysate and declare it a functional food. Additionally, investigations in animal models to elucidate the underlying mechanisms by which these peptides would exert in vivo activity and studies in humans to suggest health benefits to the peptides and their efficacy in real food matrices under specific processing and storage circumstances should be carried out. Further research with sustainable sources, such as plant proteins, is necessary in order to modify the current food system and achieve a sustainable and healthy population.

The production of bioactive peptides via enzymatic hydrolysis is a sustainable way to increase the health-promoting properties of vegetable proteins. A protein isolate obtained from chia seeds was subjected to hydrolysis with a commercially available food-grade endopeptidase in order to produce a hydrolysate with improved techno-functional and biological properties. The hydrolysate was fully characterized, including compositional data and microscopy, and the peptides were identified by LC-TIMS-MS/MS. The hydrolysate was evaluated in a transwell model employing Caco-2 cells to assess the bioavailability of the peptides and how the peptides are modified after their action. The peptides from the bioavailable fraction were also identified, showing relevant differences compared to the original hydrolysate. Twenty peptides having a molecular weight <1000 Da from this bioavailable fraction were subjected to several in silico analyses to determine their contribution to the reported bioactivity (antioxidant and ACE inhibitors) of the hydrolysate. Numerous peptides with adequate molecular features and promising scores in the prediction tools were identified and are proposed to be antioxidant and ACE inhibitor peptides from *S. hispanica* L seeds. Chia bioactive peptides have demonstrated that they can interact with the body in a multitude of ways to regulate physiological processes in different metabolic pathways throughout the organism, such as by blocking the activity of specific enzymes.

## Supporting Information Available

Molecular docking results for the peptides include the affinity of each peptide with the target ACE receptor, the cluster size, and the interaction (position, type, and distance) (Table 1) (PDF)
