# Supplementary material for: Identification of the Bioavailable Peptidome of Chia Protein Hydrolysate and the In Silico Evaluation of Its Antioxidant and ACE Inhibitory Potential
Source: J Agric Food Chem. 2024 Feb 2;72(6):3189–99. doi: 10.1021/acs.jafc.3c05331 (PMC10870759; doi:10.1021/acs.jafc.3c05331)
Supplement: Supplementary file 1 — jf3c05331_si_001.pdf [file jf3c05331_si_001.pdf]

Chia (*Salvia hispanica* L.) has proved to be a suitable source of bioactive peptides via enzymatic hydrolysis. the peptides AGDAHWTY, VDAHPIKAM, PNYHPNPR, and ALPPGAVHW are proposed to be highly contributing to the antioxidant and/or ACE inhibitors activity of the chia protein hydrolysates.
